# Is real time PCR preferable to the direct immunofluorescence in the diagnosis of *Pneumocystis jirovecii* pneumonia in HIV-infected patients?

**DOI:** 10.1186/s13104-020-05075-5

**Published:** 2020-05-01

**Authors:** Simon Bossart, Konrad Mühlethaler, Christian Garzoni, Hansjakob Furrer

**Affiliations:** 1 Department of Dermatology, University Hospital Inselspital, 3010 Bern, Switzerland; 2 Institute for Infectious Diseases, Clinical Microbiology, University of Bern, Bern, Switzerland; 3 Department of Infectious Diseases, Bern University Hospital, University of Bern, Bern, Switzerland; 4 Department of Internal Medicine and Infectious Disease, Clinica Luganese Moncucco, Lugano, Switzerland

**Keywords:** HIV, *Pneumocystis jirovecii*, Pneumocystis pneumonia, Immunofluorescence assay, Real-time PCR

## Abstract

**Objectives:**

In this study, we compared IFA and real-time PCR in bronchoalveolar lavage specimens of HIV infected patients. A total of 66 BALs from 62 HIV patients were included in the study. 30 IFA positive and 36 IFA negative specimens were tested with real-time PCR, targeting the major surface glycoprotein. We performed a retrospective analysis of the patient’s medical records, compared the results of the IFA and PCR tests and analyzed costs, expenditure of time and personal expenses.

**Results:**

All of the 30 IFA positive samples were PCR positive. 35 of 36 IFA negative probes were also negative in the PCR assay. Considering the PCR results as a binary outcome (positive/negative) sensitivity was 100%, specificity 97.2%. The patient with negative IFA and positive PCR had a clear clinical picture of PCP and responded to PCP treatment. PCR was more than twice as expensive and time-consuming as IFA. Diagnostic accuracy for PCP of PCR and IFA was comparable in HIV-infected patients, but IFA was significantly less expensive and less time-consuming. Therefore, IFA testing can continue to be used as gold standard in the diagnosis of PCP in HIV patients. However, in special cases, IFA may lack sensitivity and PCR should be added to the diagnostic armamentarium.

## Introduction

*Pneumocystis jirovecii*, an opportunistic fungal pathogen, can cause severe interstitial pneumonia (Pneumocystis pneumonia, PCP) in immunocompromised individuals (HIV and non-HIV individuals) [1, 2].

At present, in the absence of lung biopsy histology, immunofluorescence assay (IFA) of bronchoalveolar fluid (BAL) and induced sputum is considered the gold standard in diagnosing *P. jirovecii* [3].

IFA may lack of sensitivity in immunocompromised non-HIV like oncological and rheumatological patients when *Pneumocystis* load is low. In these respiratory samples IFA may be either negative or show artefacts [4]. Therefore, more sensitive PCR based methods were introduced which showed limitations in specificity. Specimens of asymptomatic immunocompromised individuals with low *Pneumocystis* load may yield a positive PCR signal while microscopic examination is negative—probably representing colonization. Notably, these discrepancies were observed in several reports in non-HIV-infected patients [4, 5] and cut-off values of quantitative PCR were tried to be established to differentiate between colonization and infection [6]. In this study we evaluate a quantitative real-time PCR for the detection of *P. jirovecii* in BAL fluids of HIV patients and compare PCR with conventional immunofluorescence assay to establish a cut-off value to distinguish between colonization and *Pneumocystis* infection. We performed retrospective chart review and compared costs, expenditure of time and personal expenses of PCR and IFA.

## Main text

### Methods

A total of 66 bronchoalveolar lavage specimen from 62 HIV patients obtained between 1998 and 2009 were enrolled in this retrospective study. 3 BAL samples from 3 patients had to be excluded in the absence of sufficient available material. The 63 BALs from the remaining 59 patients were included in our study.

The BALs were performed in the Bern University Hospital following a standardized protocol: 150 ml of sterile saline solution was instilled within the bronchial trees and recovered in three fractions. For *Pneumocystis* diagnostics, samples of 10 ml native BAL liquid were centrifuged and used for IFA diagnostics. The remaining material was frozen at −80 °C.

MONOFLUO™ *P. jirovecii* IFA Test Kit was used as gold standard for the routine diagnostics of *P. jirovecii.* The test kit consists of a commercially available murine monoclonal antibody, labeled with fluorescein isothiocynate that reacts with all forms of *Pneumocystis* stages. Specimen holders were scanned by two independent investigators with 400 × magnification in a light microscope (Zeiss Axiophot).

Semi-quantitative microscopy was performed for each sample (number of asci or trophic forms per field of vision: − = absent, + = < 1 (few), ++ = 1–10 (many), +++ = > 10 (abundant). The sensitivity of this IFA is close to 100% and the specificity is about 95.8% according to the manufacturer [7].

For the PCR procedures, nucleic acids were extracted from 25ul of BAL pellets using automated NucliSense^®^ easy MAG™ platform (bioMérieux, Switzerland). A real-time PCR was used targeting the major surface glycoprotein (MSG) gene based on the work of Linssen, 2006 [8]. The real-time PCR reaction contained 5 µl of purified DNA, 0.6 µl of each primer PCPFor and PCPRev, 0.15 µM PCPProbe, 1xTaqMan Universal Master Mix (ABI), 1x Exo IPC Mix (ABI) and 1x Exo IPC DNA (ABI). Each DNA sample was analyzed in duplication following an amplification protocol performed on an ABI PRISM 7000 Sequence Detection system (ABI).

Each cycle consists of 2 min at 50 °C (digestion of previous amplification products), 10 min at 95 °C (enzyme inactivation and polymerase activation), followed by 42 cycles of 15 s at 95 °C and 60 s at 60 °C. As positive controls different plasmid concentrations containing a *P. jirovecii* major surface glycoprotein gene (MSG) insert was used and linearized 2x10^5^, 2x10^4^, 2x10^3^, 10^3^, 2x10^2^, and 10^2^ copies per reaction to generate a standard curve. Three negative controls were included in each run. (1) water, (2) 10x Exo IPC Block (Applied Biosystems (ABI) Foster City, CA, USA; NAC = no amplification control), and a negative extraction control. In order to detect inhibitors in the specimens, an EXO IPC DNA (ABI) was included in each Real-time PCR reaction. The quantification of the *Pneumocystis* DNA was illustrated by the cycle threshold (Ct) and the number of copies/ml. Because the MSG gene consists of 50 to 100 copies, all our PCR results are based on the mean quantity with 50 copies/genome. A sample was interpreted as positive if the duplicates were positive. A retest of the sample was performed if only one result was positive. If this retest was again positive, the sample was considered positive for *P. jirovecii*. A negative sample was interpreted as confirmed negative when EXO IPC DNA (ABI) results excluded inhibitor in the specimen.

To determine the PCR detection limit of the IFA we selected a strongly IFA-positive patient with more than ten asci/trophic forms per field of vision (+++) in the semi-quantitative microscopy and performed a tenfold dilution series (log10). The detection limit was 190 copies/ml or 35 cycles. To compare the diagnostic performance of the two tests (IFA and real-time PCR), a Receiver Operating Characteristic curve analysis (ROC) was performed.

Data of the performed retrospective analysis of the patient’s medical records was analyzed by using the Stata™ 10 for Windows, StataCorp., USA: Bivariate analysis (Pearson’s Chi square test or trend Chi square), odds ratios, 95% confidence intervals and multivariate analysis were performed.

The medical history of all 62 patients were reviewed retrospectively focusing on age, sex, clinical symptoms (fever, cough, dyspnea), imaging studies, HIV background (CD4 counts, viral load, cART), laboratory analysis (LDH), PCP-Prophylaxis before BAL, PCP-Therapy after BAL and outcome. The retrospective data analysis was approved by the cantonal ethic commission of Bern.

### Results

**Fig. 1:**
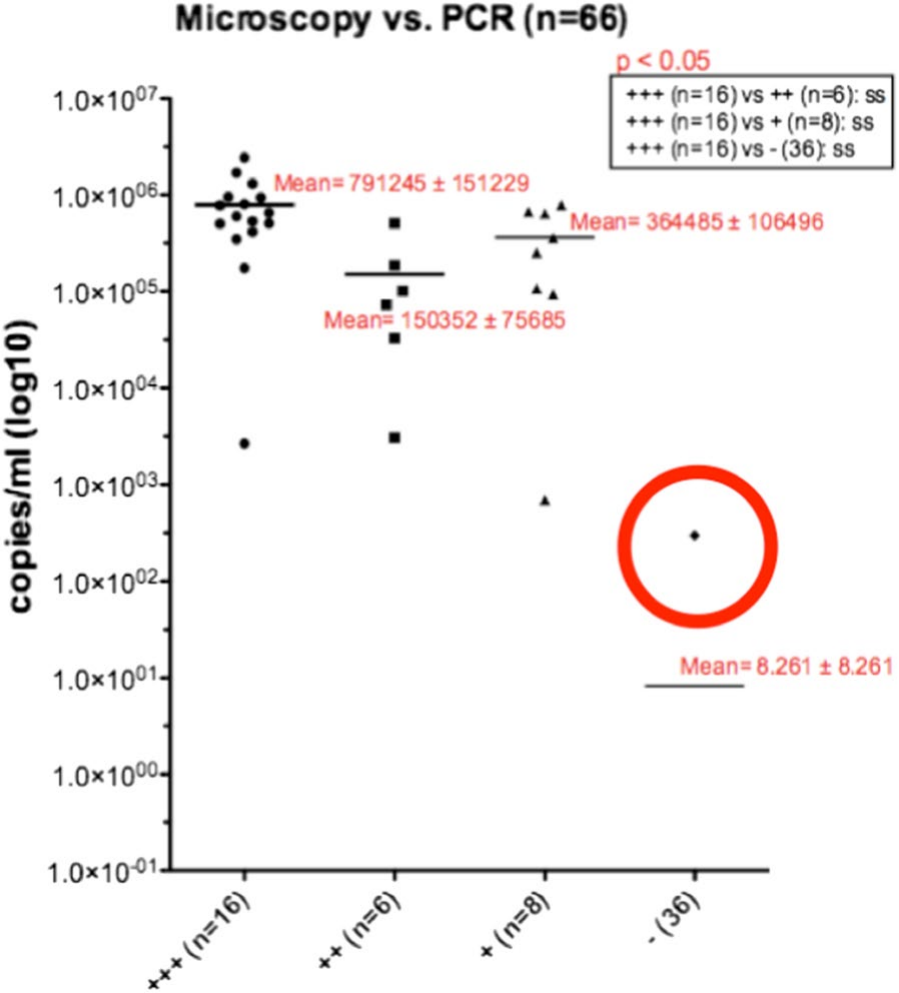
Comparison between IFA (number of asci or trophic forms per field of vision) and PCR (copies/ml) of all tested BAL samples. One specimen with a discrepant result with negative IFA and positive low copies PCR (297 copies/ml) showed in the red circle

All of the 30 IFA-positive BAL samples also tested positive by PCR. The pathogen load ranged from 698 copies/ml to 2,440,000 copies/ml. Among the 36 IFA-negative BAL probes, 35 were PCR negative. The fungal burden (number of asci or trophic formsper field of vision) correlated well with the PCR results. Only one IFA-negative BAL specimen yielded a positive PCR result. It contained the lowest *Pneumocystis* load (297 copies/ml) of all BALs tested positive (Fig. 1).

Considering the PCR results as a binary outcome (positive/negative), sensitivity was 100%, specificity 97.2%. The area under the ROC curve was 0.99.

The data of the reviewed medical history are shown in Table 1. No significant differences in age and sex were found in the IFA-negative and positive group and the clinical symptoms were similar.

**Table 1:** Detailed patient characteristics of the 62 HIV-positive patients

62 HIV positive patients	66 Bronchoalveolar lavage		Statistical significance
Characteristics	Group 1: IF positive	Group 2: IF negative	
Subjects	30	36	
Age years (median, IQR)	41 (34.5–50.5)	41.5 (33.7–48)	ns
Male/female	21/9	23/13	ns
HIV background			
CD4 + counts/ml (median, IQR)	39 (11.5– 65)	104.5 (22.7–190)	s
Viral load copies/ml (median, IQR)	189,058 (84,818.7–671,206.5)	180,009 (43,062.5–745,879.2)	ns
cART (%)	3 (10%)	26 (72.2%)	s
Symptoms between hospitalisation and BAL			
Fever	19 (63.3)	22 (61.1)	ns
Cough	25 (83.3)	32 (88.8)	ns
Dyspnea	20 (66.6)	15 (41.6)	ns
Laboratory analysis			
LDH (U/l)	588.5 (506.7–854.5)	463.5 (384.2–542.5)	s
Outcome			
In hospital death	1 (3.3)	2 (5.5)	ns
Therapy/prophylaxis			
PCP-Prophylaxis before BAL	2 (6.6)	13 (36.1)	s
PCP-Therapy after BAL	30 (100)	14 (38.8)	s
PCR Pneumocystis results			
Amount of positive qualitative PCR results	30 (100)	1 (2.7)	s
Positive quantitative PCR copies/ml (median, IQR)	506,068.59 (141,239.7–783,667.2)	298	s

Data presented as median (IQR), n (%)

*IF* immunofluorescence, *PCP* pneumocystis pneumonia, *BAL* bronchoalveolar lavage

Patients with positive IFA had similar viral loads at the last measurement before BAL, but lower CD4 counts and were less likely to be on combination antiretroviral therapy (cART). Patients with positive IFA had higher LDH levels. They were less likely to be on anti- PCP prophylaxis and more likely to be started on PCP treatment as shown in Table 1.

The single patient with the discrepant result (IFA-/PCR +) was examined in more detail. The medical history of this patient revealed that the patient had a chronic obstructive pulmonary disease (COPD) and was on antiretroviral therapy with current CD4 counts of 150 cells/µL and plasma HIV RNA of 22 copies/ml. He had the typical clinical features PCP with fever and dyspnea for several days and abnormal O2 saturation. CT scan showed marked ground glass opacities in all lung fields. In BAL no other reason for the pneumonitis was found and he responded to a 3 weeks course of trimethoprim/sulfamethoxazole. Based on this the patient was considered to have suffered of PCP.

Additional analyses concerning costs, time and personal expenses were performed. One immunofluorescence test costs CHF 23.80 while one real time-PCR is CHF 63 (ratio PCR/IFA = 2.6). Results of the IFA test is available within2 h, PCR including DNA extraction takes 4.5 h (ratio PCR/IFA = 2.25). Although IFA can easily be performed in small laboratories with a fluorescence microscope, interpretation of the IFA test may be difficult due to artefacts which can easily be mixed-up with trophic forms or asci. Therefore, skilled staff is needed to obtain correct results. For the PCR tests including DNA extraction large laboratories with accurate hardware (DNA extraction machine, thermocycler etc.) are needed but usually they are performed during day-time on working days only, limiting the real-life availability. The interpretation of the test result can be done without any special training.

### Discussion

Previous publications report a discrepancy between negative immunofluorescence and positive PCR results in immunocompromised non-HIV-infected patients with better sensitivity and specificity in the PCR assay compared to conventional, microscopic examinations [9, 10].

We could not find such a discrepancy in HIV-infected patients in the present study. We found a very good correlation in 97.2% of all 66 tested BAL specimens in 62 HIV patients. Only one specimen yielded a discrepant result: IFA was negative and PCR revealed a positive signal, corresponding to the lowest *Pneumocystis* load (298 copies/ml) of all tested BALs. The patients clinical and radiological findings and the response to PCP treatment was well compatible with PCP and based on this we did not consider this discrepant result as false positive. This patient was also known to have COPD as another lung disease. One could speculate that in this patient on virologically successful cART but CD4 counts below 200 cells/µL a lower fungal burden was associated with PCP partly also explained by COPD which has been associated with a higher risk for *Pneumocystis* colonization and higher risk of PCP in AIDS patients [11, 12]. This could explain our discrepant result.

Since we did not find false positive PCR results it was not possible to establish cut-off value for our quantitative PCR assay. In other words and in discrepancy with non-HIV immunocompromesed patients, a positive PCR with a compatible clinic should always be conceded diagnostic for PCP and lead to an appropriate therapy.

However, it must be taken into account that colonization of *Pneumocystis* can also occur in HIV-infected patients, as numerous studies have shown [13, 14].

The review of the medical records revealed lower CD4 + counts, lower rate of cART and PCP-prophylaxis and higher LDH-levels in the IFA-positive group compared with the IFA-negative specimens. Those patients have therefore stronger evidences for the presence of the infection with *Pneumocystis jirovecii.*

This study shows that both IFA and PCR have a high sensitivity for *Pneumocystis* detection in AIDS patients with PCP. Some studies found a lower sensitivity of IFA in HIV-negative individuals with PCP. These findings can be explained by the higher fungal loads in HIV-infected PCP patients compared to PCP patients with other immunodeficiencies [15, 16]. Due to the comparable diagnostic accuracy of PCR and IFA in our study, we also examined their cost-effectiveness and we could show that, in our setting, real-time PCR is three times as expensive and performance takes twice as long as the IFA assay. The benefit of the IFA is the handiness of this method that can be easily performed in small laboratories if a fluorescence microscope with appropriate filter is available. The equipment needed to run a PCR is expensive (DNA extraction machine, thermocycler etc.) and logistic issue commonly limited its use during daytime on working days. On the other hand, performing IFA requires special training while PCR does not.

### Conclusions

In summary, our findings show that PCR and IFA both are accurate diagnostic tools in HIV-infected patients. But IFA is clearly more effective regarding logistic issues and expenditure of time and cost. However, in special cases like in patients with low fungal burden and high risk for PCP, IFA may lack sensitivity. Therefore, PCR should be added in the diagnostic armamentarium of every specialized laboratory.

## Limitations

However, our study has several limitations. The data were analysed retrospectively and the study population was rather small. This has to do with the fact, that the incidence of PCP has massively declined since introduction of cART.

The fact that we did not find any colonized patients might be related to the rather small number of cases in our retrospective study. As mentioned in the discussion, larger studies have found colonization of *Pneumocystis* also in HIV positive patients.

A potential weakness of the IFA is the lack of sensitivity when fungal load is low. Therefore, interpretation of the IFA test may be difficult due to artefacts which can easily be mixed-up with trophic forms or asci.

## Data Availability

The datasets used and/or analyzed during this study are available from the corresponding author on reasonable request.

## Abbreviations

PCP: Pneumocystis pneumonia
IFA: Immunofluorescence assay
PCR: Polymerase Chain Reaction
BAL: Bronchoalveolar fluid
ROC: Receiver operating characteristic curve analysis
